# American Medical Association

**Published:** 1884-07

**Authors:** 


### Editorial.

### American Medical Association.

We are asked to give publicity to the following circular, "prepared for distribution to the various State and County Societies throughout the United States":

At the meeting of the American Medical Association held at Washington in May last, an amendment to Regulation II. was adopted, which provides that—

"Membership in the association shall be obtained by any member of a State or county medical society recognized by the association, upon application endorsed by the president of said society, and shall be retained so long as he shall remain in good standing in his local society, and shall pay his annual dues to the association."

You will perceive that, as far as such opportunities are embraced, the strength of the association will be increased and consolidated, so as to unite the profession, and give it a force and influence not otherwise attainable. Without undertaking, however, to point out the advantages of this action on the part of the association, or to advocate the plan of which it is a main feature, it may simply be said that, as the new departure has been taken, it is for the association and its constituent bodies to carry it out to the fullest extent, and to give the movement their hearty co-operation.

Toward this end, the first step is to make the action of the association as widely known as possible, and you are, therefore, requested to bring the matter to the notice of your society and its individual members, either by circular, or in such other way as may seem to you most effective for the purpose.

Applications for membership, in the manner specified above, accompanied with five dollars for annual dues, should be sent directly to the treasurer, Dr. Richard J. Dunglison, Lock Box 1274, Philadelphia, Pa., on receipt of which the weekly journal of the association will be forwarded for one year to such member.

This change we would heartily commend.

Under the old rule it was possible to throw obstacles in the way of the admission of worthy applicants for trivial or personal reasons. The adoption of the foregoing resolution puts an end to that, and also practically terminates the existence of permanent membership. Heretofore permanent members have had the privilege of paying the annual dues, but were not entitled to a vote. All from whom the dues are hereafter accepted will stand on an equal footing as active members, and as the payment also entitles them to the weekly journal of the association, the application of the resolution will undoubtedly result in a large increase in the membership, and the consequent increment of influence and effectiveness of the association.

It will, in the same proportion, increase the circulation of the "Journal," which closes the first year of its publication with its issue of June 28th. Both in a financial and literary way it has been a moderate success. Though perhaps not fully equal to the reasonable expectations of its patrons, yet it is a vast improvement upon the old method of an annual volume of "Transactions," and we have a confident hope that in the near future "The Journal of the American Medical Association" will be in the van of American journalism.

